# Predicting the Likelihood of Live Birth in Assisted Reproductive Technology According to the Number of Oocytes Retrieved and Female Age Using a Generalized Additive Model: A Retrospective Cohort Analysis of 17,948 Cycles

**DOI:** 10.3389/fendo.2021.606231

**Published:** 2021-04-30

**Authors:** Haiyan Zhu, Chenqiong Zhao, Peiwen Xiao, Songying Zhang

**Affiliations:** ^1^ Assisted Reproduction Unit, Department of Obstetrics and Gynecology, Sir Run Run Shaw Hospital, School of Medicine, Zhejiang University, Hangzhou, China; ^2^ Department of Obstetrics and Gynecology, Key Laboratory of Reproductive Dysfunction Management of Zhejiang Province, Hangzhou, China; ^3^ School of Statistics, Jiangxi University of Finance and Economics, Nanchang, China

**Keywords:** female age, ovarian stimulation, cumulative live birth rate, predictive model, oocyte numbers

## Abstract

**Capsule:**

We designed a predictive reference model to evaluate how many stimulation cycles are needed for a patient to achieve an ideal live birth rate using assisted reproductive technology.

**Objective:**

To develop a counseling tool for women who wish to undergo assisted reproductive technology (ART) treatment to predict the likelihood of live birth based on age and number of oocytes retrieved.

**Methods:**

This was a 6-year population-based retrospective cohort analysis using individual patient ART data. Between 2012 and 2017, 17,948 women were analyzed from their single ovarian stimulation cycle until they had a live birth or had used all their embryos. All consecutive women between 20 and 49 years old undergoing their ovarian stimulation cycles for ART in our center were enrolled. The cumulative live birth rate (CLBR) was defined as the delivery of a live neonate born during fresh or subsequent frozen–thawed embryo transfer cycles. Only the first delivery was considered in the analysis. Binary logistic regression was performed to identify and adjust for factors known to affect the CLBR independently. A generalized additive model was used to build a predictive model of CLBR according to the woman’s age and the number of oocytes retrieved.

**Results:**

An evidenced-based counseling tool was created to predict the probability of an individual woman having a live birth, based on her age and the number of oocytes retrieved in ART cycles. The model was verified by 10 times 10-fold cross-validation using the preprocessed data, and 100 area under the curve (AUC) values for receiver operating characteristic (ROC) curves were obtained on the test set. The mean AUC value was 0.7394. Our model predicts different CLBRs ranging from nearly 90% to less than 20% for women aged 20–49 years with at least 22 oocytes retrieved. The CLBRs of women aged 20–28 years were very similar, nearly on one trend line with a certain number of oocytes retrieved. Differences in the CLBR began to appear by the age of 29 years; these increased gradually in women aged >35 years.

**Conclusion:**

A predictive model of the CLBR was designed to serve as a guide for physicians and for patients considering ART treatment. The number of oocytes needed to be retrieved to achieve a live birth depends on the woman’s age.

## Introduction

Controlled ovarian stimulation is a key component of assisted reproductive technologies (ARTs) and it has resulted in a significant increase both in the numbers of frozen–thawed embryo transfers and in pregnancy rates (1, 2). Traditionally, the success of ART has been reported as clinical or ongoing pregnancy rates, implantation rates, and live birth per embryo transfer. However, the cumulative live birth rate (CLBR), defined as the first live birth following the use of all embryos from one complete cycle including fresh and subsequent frozen–thawed cycles appears to be a better measure of ART treatment success (3, 4). The CLBRs increase with the number of oocytes retrieved, reportedly reaching as high as 70% when ≥25 oocytes are retrieved, with no plateau in CLBRs (5). Although ART treatment allows for multiple follicular development and can help produce multiple embryos, there is no consensus on the optimal number of oocytes needed to retrieve and the CBLR for each individual women after each stimulated *in vitro* fertilization (IVF) or intracytoplasmic sperm injection (ICSI) ART cycle.

It is well known that female age is the main factor affecting the success rate of ART. Ovarian function and the quality of retrieved oocytes are inherently linked with female age, with older women being less likely to obtain many oocytes retrieved and with higher risk of developing oocytes and embryos with aneuploidy (6–8). Consequently, it is reasonable to assume that women of advanced age will require more oocytes to be retrieved to achieve a live birth than younger women. Nevertheless, in clinical practice, clinicians often encounter elderly women wishing to obtain advice about their chances of getting a live birth. There are even many women who have underwent one or two stimulation cycles, and have frozen one or two embryos, who come to consult whether they need to undergo another stimulation cycle to obtain more oocytes, thus increasing the probability of live birth. Moreover, even among similarly aged young women, the numbers and quality of oocytes obtained per stimulation cycle vary widely (9, 10). Therefore, it is difficult to predict how many oocytes each woman should obtain to optimize her probability of having a live birth. Furthermore, in addition to increasing the success rate as much as possible for patients during ART treatment, it is also necessary to reduce the risk to patients as much as possible, so appropriate individualized stimulation protocols are needed. The target number of oocytes retrieved should be set according to the patient’s age, ovarian reserve and clinical condition. The possible drawbacks for patients after large number of oocytes retrieved are considerable pain and discomfort, an increased risk of bleeding, adnexal torsion predisposed by enlarged ovaries, and a slight increase in the rare incidence of thromboembolic events caused by high estradiol (E2) levels (11). Consequently, young patients may need to reduce the dose of gonadotropins or switch to micro stimulation, which could help avoid the occurrence of ovarian hyperstimulation syndrome (OHSS) and achieve an ideal success rate. However, the accumulation of cryopreserved oocytes or embryos over several cycles might be needed by older women to improve the CLBR. Thus, questions that often arise in clinical practice are as follows. How many oocytes are sufficient to achieve a live birth for each woman? How many ART cycles are required for older women to increase their CLBR?

Here, we aimed to develop a counseling tool for women wishing to undergo ART treatment and help predict the likelihood of achieving at least one baby based on the woman’s age and the number of oocytes retrieved. The CLBR following utilization of all fresh and frozen embryos in women undergoing a single ovarian stimulation cycle were also evaluated. This model could be used to guide women and their physicians regarding the estimated number of oocytes required and also to inform women whether undergoing additional stimulation cycles would result in a meaningful increase in their CLBR.

## Materials and Methods

### Study Design

We conducted a retrospective analysis of 17,948 fresh IVF/ICSI cycles performed from January 2012 to December 2017 in the Centre for Reproductive Medicine of the Sir Run Run Shaw Hospital of Zhejiang University. This study was approved by the institutional review board of our hospital.

### Patients’ Eligibility Criteria

**Figure 1:**
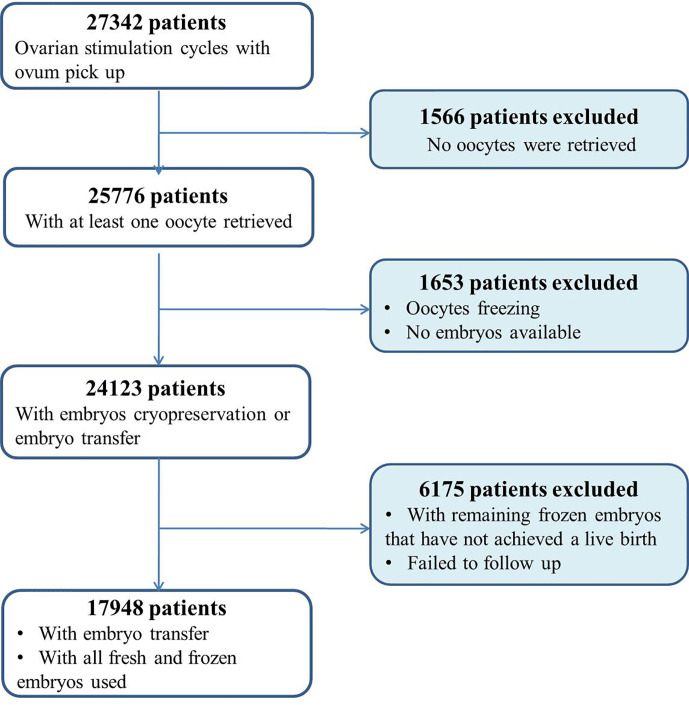
Flow chart of patient selection and exclusions.

Eligible patients were considered as all consecutive women between 20 and 49 years of age undergoing ovarian stimulation cycles for IVF/ICSI in our center. Cycles with preimplantation genetic testing or medically indicated cryopreservation of oocytes were excluded. Women with no oocytes retrieved were also excluded. In addition, women who did not deliver a live baby but who still had frozen embryos remaining were excluded from the analysis (**Figure 1**). Only these women who either delivered a baby or used all their embryos after a single stimulation cycle were included. The length of follow-up was 3 years.

### Treatment Protocol

Patients were treated by recombinant follicle stimulating hormone and/or urinary human menopausal gonadotropins (rFSH/hMG) in ovarian stimulation protocols followed by IVF or ICSI. The stimulation protocol and rFSH/hMG doses were chosen depending on age, ovarian reserve tests and basal follicle numbers. Follicular development was monitored by ultrasonography, and final oocyte maturation was induced by injection of human chorionic gonadotrophin (hCG) when at least three follicles of 18 mm in diameter were observed. Oocyte retrieval was performed 36 h after hCG administration. For luteal phase support, intramuscular injections of progesterone (60 mg qd) combined with oral progesterone (20 mg qd) were started the day after oocyte retrieval, reduced to intramuscular injection progesterone (40 mg qd) combined with oral progesterone (20 mg qd) after a positive beta-hCG pregnancy test and until 8 weeks of pregnancy. Embryo quality in culture was assessed according to number of blastomeres and percentage of fragmentation as reported (12). An embryo with 6–12 blastomeres graded 1 and 2 was defined as good quality.

Fresh embryo transfer was carried out under ultrasound guidance 3 or 5 days after oocyte retrieval. Supernumerary day 3 embryos with at least four blastomeres and ≤35% fragmentation were selected for cryopreservation using vitrification protocols.

For frozen embryo transfer (FET) cycles, embryos were evaluated for morphological survival immediately after warming. Embryos with at least 50% of their cells intact were considered as having survived and being suitable for transfer. Three main types of clinical protocols were used for endometrial preparation: natural, hormone replacement, or human menopausal gonadotropin (HMG)-stimulated cycles (12). The type of preparation for the FET cycle was based on the individual menstrual cycle pattern of the patient.

### Main Outcome Measures

The primary outcome was the CLBR defined as the delivery of a live neonate in the fresh or in the subsequent frozen–thawed cycles in relation to the woman’s age and the numbers of oocyte retrieved. Only the first delivery was considered in the analysis for CLBR.

### Statistical Methods

Continuous data are presented as the mean value ± standard deviation (SD) and were compared using Student’s *t* tests. Categorical data are described as the number of cases and percentages and were analyzed using Pearson’s Chi-squared test. Patients were categorized into two groups according to the outcome: CLBR or no live birth. Patients’ baseline characteristics and ovarian stimulation characteristics of the IVF/ICSI treatment were also analyzed. A multivariate logistic regression using binary logistic regression was performed to assess the association between the numbers of oocytes retrieved and the CLBR, after adjustment for relevant confounders. The potential predictors considered for the analysis were the woman’s age, duration of infertility, stimulation protocol, duration of stimulation, fertilization rate, number of good quality embryos, and insemination method (IVF or ICSI). The regression models are presented as the odds ratio (OR) with standard error (SE) and 95% confidence interval (CI). Analyses were conducted using IBM SPSS Statistics (v. 22.0; IBM Corp., Armonk, NY, USA). In addition, a predictive model of CLBR according to the woman’s age and numbers of oocytes retrieved was built. A Generalized Additive Model was applied using RStudio software (RStudio, Boston, MA, USA; package mgcv) to calculate the CLBR depending on the woman’s age and numbers of oocyte retrieved.

## Results

### Patient Characteristics

In total, 17,948 patient cycles were analyzed. The patients’ baseline characteristics and ovarian stimulation characteristics are presented in **Table 1**. Among them, 11,121 (62.0%) patients achieved a live birth either in the fresh or in subsequent FET cycles. Of these, 6,827 (38.0%) patients did not achieve a live birth. First, through descriptive analysis, we found that compared with the group without a live birth, the mean age was 2.7 years younger, and the number of oocytes retrieved was 4.3 more in the group with a live birth (**Table 1**; **Supplemental Figure S1**). Moreover, comparisons between patients who did or did not achieve live birth revealed significant differences for duration and infertility and basal ovarian function (menstrual Day 3 E2 and FSH levels). The stimulation protocols, duration of stimulation, type of insemination method, fertilization rate, good quality embryo rate, and number of day-3 embryo transferred in the fresh cycle were also significantly different between the two groups. Higher fertilization rates, numbers of good quality embryos and the numbers of embryos to cryopreserve were observed in patients who achieved a live birth. No significant differences were noticed for the indication of infertility or day of embryo transfer in fresh cycles. The majority of patients (>90%) transferred the day 3 embryos in both two groups.

**Table 1:** Basal characteristics and fresh/FET IVF/ICSI outcomes.

	Live birth	No live births	*P*	OR (95% CI)
No. of stimulation cycles, n (%)	11,121 (62.0%)	6,827 (38.0%)		
Age (mean ± SD)	30.5 ± 3.8	33.3 ± 5.1	<0.001	
D3 FSH (IU/ml) (mean ± SD)	7.0 ± 2.4	7.9 ± 3.2	<0.001	
D3 E2 (mean ± SD)	40.4 ± 27.9	43.0 ± 28.4	<0.001	
Infertility factor, n (%)			0.069	
Tubal factor	7,162 (64.4%)	4,488 (65.7%)		0.943 (0.885–1.004)
Endometriosis	463 (4.2%)	314 (4.6%)		
Ovulatory disorder	508 (4.6%)	171 (2.5%)		
Diminished ovarian reserve	377 (3.3%)	517 (7.6%)		
Male factor	1,944 (17.5%)	941 (13.8%)		
Unexplained	667 (6.0%)	396 (5.8%)		
Duration of infertility (years) (mean ± SD)	3.4 ± 2.6	4.1 ± 3.2	<0.001	
Stimulation protocol, n (%)			<0.001	
Long protocols	6,638 (59.7%)	2,543 (37.2%)		2.494 (2.344–2.654)
Short protocols	1170 (10.5%)	1121 (16.4%)		
Ultralong protocols	981 (8.8%)	641 (9.4%)		
Micro-stimulation protocols	575 (5.2%)	1,081 (15.9%)		
Others	1,757 (15.8%)	1,441 (21.1%)		
Duration of stimulation (day) (mean ± SD)	9.8 ± 1.9	9.1 ± 2.5	<0.001	
No. of oocytes retrieved (mean ± SD)	11.4 ± 6.7	6.9 ± 5.0	<0.001	
Use of ICSI, n (%)	3,631 (32.6%)	2,718 (39.8%)	<0.001	0.733 (0.688–0.780)
Fertilization rate (%)				
IVF	70.60%	66.20%	<0.001	1.223 (1.189–1.259)
ICSI	77.20%	73.60%	<0.001	1.211 (1.161–1.263)
Good quality embryo rate (%)	49.80%	41.10%	<0.001	1.422 (1.385–1.460)
No. of good quality embryo (mean ± SD)	4.2 ± 8.0	2.3 ± 1.7	<0.001	
No. of frozen embryo (mean ± SD)	6.7 ± 4.4	3.6 ± 2.7	<0.001	
Day of embryo transfer in the fresh cycle				
Day-3 embryo transfer, n (%)	1476 (92.7%)	1,152 (93.6%)	0.37	0.865 (6.44–1.163)
Blastocyst transfer, n (%)	117 (7.3%)	79 (6.4%)	0.37	
No. of embryo transferred in the fresh cycle				
No. of day-3 embryos transferred (mean ± SD)	1.8 ± 0.4	1.7 ± 0.5	<0.001	
No. of blastocysts transferred (mean ± SD)	1.4 ± 0.5	1.5 ± 0.5	>0.05	
Day of embryo transfer in the frozen cycle				
Day-3 embryo transfer, n (%)	10,556 (85.1%)	6,666 (84.0%)	0.028	1.092 (1.010–1.180)
Blastocyst transfer, n (%)	1,842 (14.9%)	1,270 (16.0%)	0.028	
No. of embryo transferred in the frozen cycle				
No. of day-3 embryos transferred (mean ± SD)	1.9 ± 0.4	1.8 ± 0.5	<0.001	
No. of blastocysts transferred (mean ± SD)	1.6 ± 0.5	1.5 ± 0.5	<0.001	

FSH, follicle stimulating hormone; E2, estradiol; IVF, in vitro fertilization; ICSI, intracytoplasmic sperm injection; OR, odds ratio; 95% CI, 95% confidence interval.

### Associations Between the Number of Oocytes and CLBR for Each Age Group

A generalized additive model was developed after variables selection and parameter adjustment. Among all the model variables, the woman’s age and the number of oocytes retrieved had the greatest influence on the model. Using this model, a binary prediction of cumulative live birth was established based on the original data. Because there are categorical variables in the prediction variables, these were treated as dummy variables in the model. These were introduced into the model, and the rest of the numerical variables were treated as smooth functions. The model was verified by 10 times 10-fold cross-validation using the preprocessed data, and 100 area under the curve (AUC) values for receiver operating characteristic (ROC) curves were obtained on the test set. The mean AUC value was 0.7394 (**Supplemental Figure S1**), which indicates that in clinical practice, it is necessary to combine the specific physical conditions of couples, and then produce a judgment according to our model.

**Figure 2:**
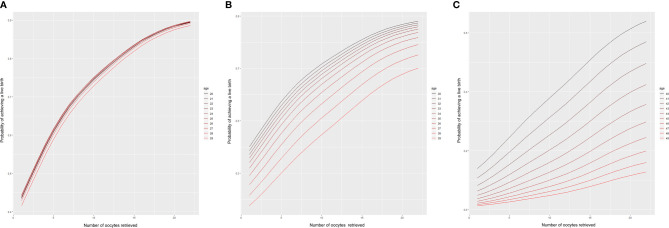
Predictions of the CLBR according to age and the number of oocytes retrieved. Each curve represents the likelihood that a patient of a given age will obtain a live birth based on the number of oocytes retrieved. **(A)** Patients aged 20–29 years; **(B)** patients aged 30–39 years; **(C)** patients aged 40-49 years.

The variables of woman’s age and number of oocytes retrieved had large influences in the whole model; therefore, the values of other variables were set as the average values. A linear trend chart using the number of oocytes retrieved and the CLBR was constructed according to the woman’s age range of 20–50 years. The 95% quantile of the number of oocytes retrieved in the original data was 22. Consequently, the number of oocytes retrieved on the horizontal axis of linear trend chart corresponds to 022, and the vertical axis represents the corresponding live birth rates. The probability of having a live birth as a function of the number of oocytes retrieved is shown in **Figure 2**.

According to the model, we found that among women aged 20–28 years, the cumulative live rate was very similar, nearly on one trend line for a certain number of oocytes retrieved. When more than 22 oocytes were retrieved, the CLBR was as high as 90%. Even when the number of oocytes retrieved was only about 10, women aged 20–28 years old could be expected to have a 75% likelihood of having a live birth. However, the CLBR began to diverge between age groups after the age of 29. The older the women, the lower the cumulative live birth rate. Moreover, the difference gradually increased in those older than 35 years. With 20 oocytes retrieved, the cumulative live birth rate decreased gradually from nearly 80% at 35 years of age to less than 70% at 39 years. For women aged 40 years, even with 20 oocytes retrieved, the CLBR was only about 60%. Furthermore, it dropped to less than 40% for women aged >43 years.

### Multivariate Logistic Regression Analysis for the CLBR

To control for possible confounding variables that might have changed over the study period, multivariate logistic regression analysis was applied to estimate the OR with the SEs and 95% CI of CLBRs for the variables age and numbers of oocytes retrieved. As shown in **Table 2**, after adjustment for the duration of infertility, insemination method, fertilization rates, and number of good quality embryos, the age and number of oocytes remained independent predictive factors (*P* < 0.001). The OR of CLBRs for age was 0.902 (95% CI 0.893–0.910), for the number of oocytes was 1.036 (95% CI 1.022–1.050). The duration of infertility and insemination method did not show any significant effect on the CLBR.

**Table 2:** Logistic regression analysis with odds ratios for CLBR.

Cumulative live birth	Odds ratio	SE	95% CI	*P*
Age	0.902	0.005	0.893–0.910	<0.001
Oocytes	1.036	0.007	1.022–1.050	<0.001
Good quality embryo	1.362	0.115	1.088–1.705	0.007
Fertilization rate	1.265	0.009	1.234–1.296	<0.001
Insemination method				
IVF	1.077	0.04	0.996–1.165	0.062
ICSI	1			
Duration of infertility	0.989	0.007	0.975–1.003	0.109

CLBR, cumulative live birth rate; 95% CI, 95% confidence interval; SE, standard error of the mean.

## Discussion

As the number of women who require ART treatment increases steadily in China, so does the demand for individual counseling tools that can build realistic expectations and accurately predict their probabilities of having a live birth. Frequently, patients will ask about the ‘optimum’ number of oocytes retrieved and the exact chance of achieving a live birth. Of course, this is challenging to estimate as it depends on a number of factors, including maternal and paternal age and health, maternal oocyte quality, and paternal sperm quality. This study provides a model to predict a patient’s probability of having a live birth based on her age and the number of oocytes retrieved. Furthermore, it also offers the clinicians a predictive reference model to evaluate how many stimulation cycles are needed for a patient to achieve an idea CLBR and gives the patient a psychological preparation and anticipation.

Based on our model, the CLBRs increase continuously with the number of oocytes retrieved in all age categories, and lower CLBRs are seen with increasing age for a given number of oocytes retrieved. The number of oocytes retrieved and the woman’s age proved to be very important variables independently associated with the CLBR. Overall, for each age category, the more oocytes retrieved the better the ART treatment outcome, consistent with previous analyses by Law et al. (1) and Polyzos et al. (5). This is reasonable as more oocytes retrieved can lead to supernumerary embryos, which could further increase the CLBR, and this was reflected in our results. Those patients who achieved a live birth had more oocytes retrieved, better fertilization rates, and more embryos available to cryopreserve compared with those who did not achieve a live birth. Furthermore, our study showed that the number of oocytes retrieved was positively associated with the number of good quality embryos, so that higher good quality embryo rates were observed in patients who achieved a live birth. In complete contrast to the results from the current study, previous studies investigating the relationship between the number of oocytes retrieved and embryo quality have suggested that high oocyte yields can actually *reduce* embryo quality (13, 14). Nevertheless, Vaughan et al. demonstrated a positive relationship between the number of blastocysts and the number of oocytes retrieved (15). Furthermore, although no consensus was reached in the relationship between the number of oocytes retrieved and the percentage of euploid embryos, the final total number of euploid embryos does increase with the number of oocytes (16–18), which explains the increased CLBRs in the higher oocyte number categories as arising from a higher availability of euploid embryos available for transfer.

Several studies have been proposed to investigate the relationship between the number of oocytes retrieved and the CLBR (1, 5, 19–21). In agreement with our results here, those studies also found a positive association between oocyte retrieved and cumulative live birth rates. Nevertheless, all these studies analyzed the cumulative live birth rates *via* categorizing patients into several groups according to age and the number of oocytes retrieved. None of them focused on the probability of live birth rates for each age group after retrieving different numbers of oocytes. Thus, Devesa et al. only enrolled women aged ≥38 years (19). Patients in the study carried out by Law et al. were stratified into five groups (1). The live birth rates of patients aged 18–34 years reached a plateau after ≥25 oocytes were retrieved. In contrast, in our study no such plateau in the CLBR was observed. Polyzos et al. retrospectively reviewed the CLBRs of 14,469 women undergoing ovarian stimulation (5). In that study, a logistic regression model was created to assess the association between the number of oocytes and the CLBR for women aged 18–45 years. The authors found that the CLBR increased steadily with the number of oocytes, reaching 70% when ≥25 oocytes were retrieved. By contrast, our model predicts different CLBRs from nearly 90% to less than 20% for women aged 20–49 years with 22 oocytes retrieved. Although our results are similar to those of Law et al. and Polyzos et al., the differences may be driven by our finer stratification of women’s age by yearly increments rather than into age groups. Furthermore, our study differs from these registry analyses, possibly because of the application of a “freeze-all” embryos policy in most cases. In our study, the rate of freeze-all with subsequent FET reached nearly 85%, and only 15% of the cases underwent fresh embryo transfer. A considerable benefit could be seen from FET, as the pregnancy rates in our center are as high as 58% (12). Meanwhile, vitrification was introduced in our ART center in 2012; the 97% embryo survival rate maximizes the utilization of oocytes retrieved, which increased the live birth rate per oocyte retrieved.

Differences in modeling methodology make direct comparisons between current study and previous studies. As previous studies analyzed the cumulative birth rates by age stratifications rather than by individual age, or by number of oocyte retrieved rather than individual number of oocyte retrieved, this might have over- or under-estimated the likelihood of live birth. Here, we included a complete population of women aged 20–49 years, predicting how many oocytes they would need to retrieve to achieve a certain live birth rate at each individual age, or how the CLBR could be achieved by retrieving a certain number of oocytes at each individual age. Our results suggest that the number of oocytes needed differed in women of different ages, as the expected probability of a live birth from maximizing the oocyte numbers retrieved decreased with advancing age. According to our model, although the CLBRs reached gratifying levels of almost 90% in women aged 20–28 years and 70% for women aged 39 years when 22 oocytes were retrieved, a clinically significant decline of nearly 50% was identified with women aged ≥44 years. This is understandable, as embryo aneuploidy rates increase with age which would have decreased the live birth rate.

Of note, this model was designed to help predict the CLBR for infertile women desiring ART treatment. It can be used during initial consultations to help provide reasonable expectations regarding the number of oocytes retrieved potentially needed for achieving a live birth. Moreover, it helps patients, especially those of advanced age patients, to decide how many stimulation cycles to undergo and to recognize that a 100% likelihood of live birth is not guaranteed. While the predictive model generated from our analysis was specifically based on the outcomes of fresh and frozen cycles in our center, this model would overestimate the probability of live birth if a woman was to be treated at a center with lower success rates. As our model was based on aggregated data of a 97% survival of thawed embryos and a 58% pregnancy rate for FET cycles, variations between studies and centers would clearly impact on the outcomes (12). Thus, individual IVF centers might need to modify our model based on their own embryo thaw survival, pregnancy rates and live birth rates, thereby offering more specific counseling for their patients in their own centers.

However, there were limitations with our study that need to be highlighted. First, our model was based on a retrospective study over a long period, and it is possible clinical practice might have changed. The retrospective study design is associated with inherent biases that may affect our results. Although we tried to minimize bias by controlling for a larger number of known confounders, we still cannot exclude the presence of unknown confounders which could not be adjusted in our model. In addition, potential confounders such as anti-Müllerian hormone levels and body mass index were not measured and so were unavailable for analysis in our study. Finally, we did not consider male factors in our model. It has long been realized that sperm quality can affect the embryo quality and development. In this regard, our model may have reduced accuracy for couples with male factor infertility such as teratozoospermia. Consequently, the AUC value of the ROC curve in our model is 0.7394, which indicates that in clinical practice, it is necessary to combine the specific physical conditions of couples and then give the judgment according to our model.

In conclusion, this live birth predictive model for the CLBR was designed to serve as a guide for women considering ART treatment. The number of oocytes needed to be retrieved for achieving live birth depends on female age. The higher the oocyte number retrieved, the higher the probability of achieving a live birth. As this study is based on retrospective data, our model should be verified with a prospective study in the future.

## Data Availability Statement

The original contributions presented in the study are included in the article/**Supplementary Material**. Further inquiries can be directed to the corresponding authors.

## Ethics Statement

The studies involving human participants were reviewed and approved by the institutional review board of Sir Run Run Shaw hospital. The patients/participants provided their written informed consent to participate in this study.

## Author Contributions

HZ and SZ designed the study. HZ and CZ drafted the article. PX and HZ performed the statistical analysis. All authors contributed to the article and approved the submitted version.

## Funding

This work was supported by grants from National Key Research and Development Program of China (2018YFC1004800), the Natural Science Program of Zhejiang (LY19H040009), and the National Natural Science Foundation of China (No. 81601236).

## Conflict of Interest

The authors declare that the research was conducted in the absence of any commercial or financial relationships that could be construed as a potential conflict of interest.

## Supplementary Material

Supplementary Figure 1 The distribution of patients who did or did not achieve a live birth and the ROC curve of the predictive model. **(A)** The distribution of patients who did or did not achieve a live birth based on the number of oocytes retrieved. **(B)** The distribution of patients who did or did not achieve a live birth based on age. **(C)** The ROC curve of the predictive model; the AUC value of ROC curve is 0.7394.
