# Supplementary figures and images for: Predicting the Likelihood of Live Birth in Assisted Reproductive Technology According to the Number of Oocytes Retrieved and Female Age Using a Generalized Additive Model: A Retrospective Cohort Analysis of 17,948 Cycles
Source: Front Endocrinol (Lausanne). 2021 Apr 30;12:606231. doi: 10.3389/fendo.2021.606231 (PMC8120808; doi:10.3389/fendo.2021.606231)

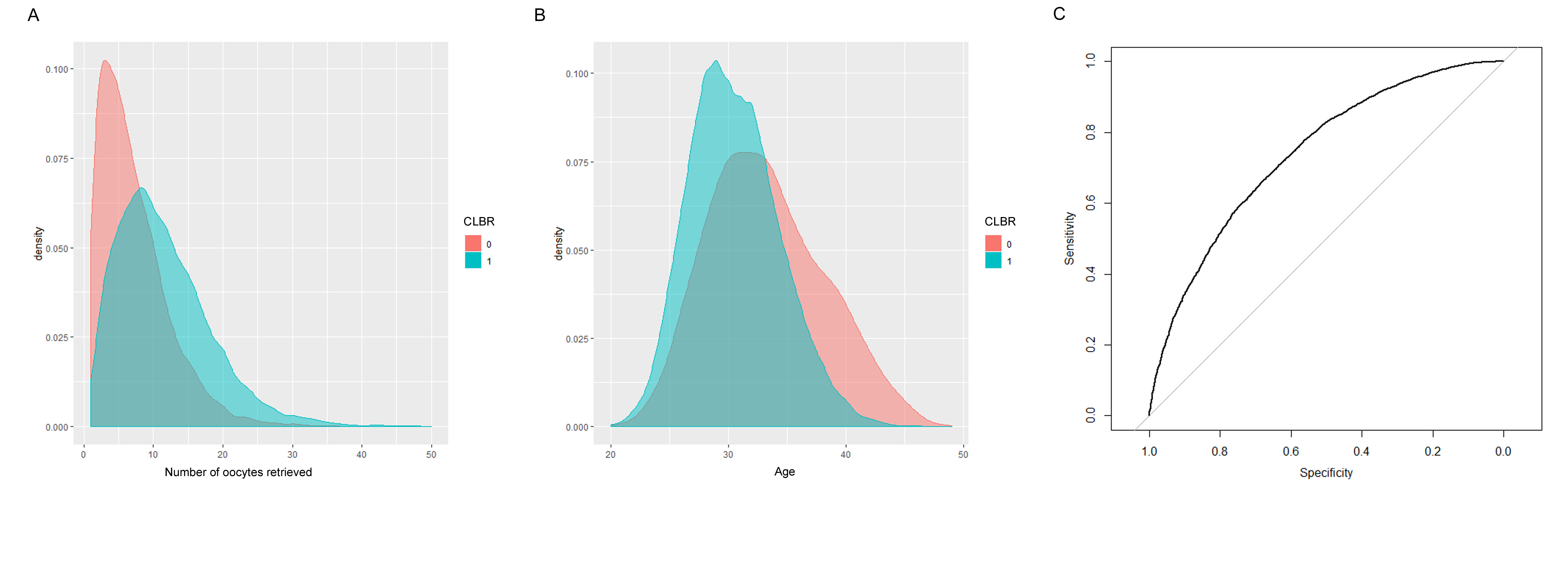

Supplement: Supplementary Figure 1 — The distribution of patients who did or did not achieve a live birth and the ROC curve of the predictive model. (A) The distribution of patients who did or did not achieve a live birth based on the number of oocytes retrieved. (B) The distribution of patients who did or did not achieve a live birth based on age. (C) The ROC curve of the predictive model; the AUC value of ROC curve is 0.7394. [file Image_1.tif]
